# How effective is indocyanine green (ICG) in localization of malignant pulmonary nodules? A systematic review and meta-analysis

**DOI:** 10.3389/fsurg.2022.967897

**Published:** 2022-07-25

**Authors:** Andreas Gkikas, Savvas Lampridis, Davide Patrini, Peter B. Kestenholz, Marco Scarci, Fabrizio Minervini

**Affiliations:** ^1^ Department of General Surgery, Hillingdon Hospital, The Hillingdon Hospitals NHS Foundation Trust, London, United Kingdom; ^2^ Department of Thoracic Surgery, Guy’s and St Thomas’ NHS Foundation Trust, London, United Kingdom; ^3^ Department of Thoracic Surgery, University College London Hospitals, London, United Kingdom; ^4^ Department of Thoracic Surgery, Cantonal Hospital Lucerne, Lucerne, Switzerland; ^5^ Department of Thoracic Surgery, Imperial College Healthcare NHS Trust, London, United Kingdom

**Keywords:** Indocyianine green, ICG, pulmonary nodules, lung surgery, lung malignancy

## Abstract

**Background:**

Video-Assisted and Robotic-Assisted techniques become constantly more prominent practice in thoracic surgery for lung cancer. Furthermore, the increased frequency in detection of small lung cancers makes the intra-operative identification of these cancers even more challenging. Indocyanine Green (ICG) is one of the most commonly used dyes that assists surgeons identify small lung cancers intra-operatively. Our study aimed to evaluate the effectiveness and safety of ICG in lung cancer detection.

**Methods:**

We performed a systematic review of the literature by screening the databases of MEDLINE, EMBASE, CENTRAL and Scopus until 30th April 2022 and the first 300 articles of Google Scholar for any suitable grey literature. We included any study that investigated the effectiveness of ICG in lung cancer detection. We excluded studies that explored the use of ICG only in identification of intersegmental planes, lymph node mapping, case reports and non-English articles. We aimed to perform a meta-analysis on test accuracy studies using hierarchical summary receiver operating characteristic (HSROC) and the bivariate random-effects models. In cases where the data for a localization technique was not sufficient for that analysis, it was presented with tables with narrative purposes. Each study was assessed for Risk of Bias (RoB) and Applicability using the QUADAS-2 tool.

**Results:**

We found 30 eligible studies that included a total of 1,776 patients who underwent ICG localization of pulmonary nodules. We identified three ICG localization techniques: CT-guided, endobronchial and intravenous. From the 30 studies, 13 investigated CT-guided localization, 12 explored an endobronchial method while 8 studies administered ICG intravenously the median reported success rate was 94.3% (IQR: 91.4%–100%) and 98.3% (IQR: 94%–100%) for the first two techniques respectively. Intravenous ICG lung cancer localization showed Sensitivity of 88% (95% CI: 59%–0.97%) and Specificity of 25% (95% CI: 0.04%–0.74%). There were 15.2% (150/989) patients who experienced complications from CT guided ICG localization. No ICG-related complications were reported in endobronchial or intravenous techniques.

**Conclusion:**

Our study provides a comprehensive review of the literature on ICG localization techniques for lung cancer. Current evidence suggests that ICG is boh effective and safe. Further prospective research with standardized protocols across multiple thoracic units is required in order to accurately validate these findings.

## Introduction

Lung cancer remains one of the leading causes of mortality globally, accounting for almost a quarter of all cancer related deaths, even in developed counties (1, 2). Computer Tomography (CT) scan is considered the gold standard imaging modality for detection of even sub-centimeter lung nodules (3). Recent evidence demonstrates lower lung cancer mortality among high risk population who undergo volume-based, low-dose computed tomographic (CT) lung cancer screening program (4). Furthermore, the screening program detected higher rate of early stage lung cancers, compared to control, which subsequently offers treatment sooner to smaller pulmonary nodules with surgery being a key component (4).

The latest evidence from VIOLET trial supports that minimally invasive techniques offer more favorable results compared to the open approach (5). Furthermore, arising evidence suggests that anatomically sub-lobar resections, such as segmentectomy, provides better outcome compared to lobectomy in early stage, peripheral lung cancer (6). This evidence drives a clear trend in thoracic surgery over the last decade in resecting constantly smaller lung nodules (7–9). Despite the high quality cameras available in Video-Assisted Thoracoscopic Surgery (VATS) and Robotic-Assisted Thoracoscopic Surgery (RATS), the pre-operatively identified sub-centimeter ground glass opacities with small percentage of solid component that are located at a distance from the lung surface make their intra-operative identification a rising challenge (7–9). For that reason, multiple techniques for lung cancer localization have been developed in attempt to address this rising challenge (9, 10).

Indocyanine Green (ICG) is one of the most common localization techniques for early stage lung cancer. ICG is a near-infrared (NIR) fluorescent contrast agent with fluorescence absorption of 820 nm which is visible with appropriate cameras (11). NIR light provides high tissue penetration with low autofluorescence which makes it useful for contrast (11).

Despite a number of narrative reviews highlighting the usefulness of indocyanine green (ICG) in thoracic surgery, its true value remains equivocal (12–14). For that reason, we conducted a systematic review of the literature that would investigate the effectiveness and safety of ICG in detection of malignant lung nodules.

## Materials and methods

### Team set up and study selection criteria

To complete this project according to the guidance for systematic review and meta-analysis we set up a research team. After discussions amongst team members, we designed our research protocol which was published in PROSPERO (CRD42022338004). The results of the study were reported following the Preferred Reporting Items for Systematic Reviews and Meta-Analyses (PRISMA) guidelines (PRISMA Checklist is provided in the Appendix).

We concluded on the inclusion and exclusion criteria below:

Inclusion Criteria:

•  Patients undergoing resection of pulmonary nodules following the use of indocyanine green (ICG) with near-infrared fluorescence imaging (NIR).
•  All surgical approaches (open, video-assisted or robotic-assisted thoracoscopic surgery).

Exclusion Criteria:

•  Overlapping patient cohorts (inclusion of the latest study only to avoid duplication of data).
•  Studies that investigate the use of ICG only for identification of intersegmental planes.
•  Studies that investigate sentinel lymph node mapping.
•  Articles that contribute no data for analysis (reviews, commentaries, editorials, etc.).
•  Case reports.
•  Language other than English.
•  Full text unavailable.

### Search strategy

To achieve a broad and inclusive review of the literature we used only keywords relevant to the disease of lung cancer, the surgical approach and ICG.

This systematic review is comprised of studies that investigate the effectiveness of peri-operative indocyanine green (ICG) administration in the detection of malignant pulmonary nodules. There was no limitation with regards to the year of publication, method of ICG administration or surgical approach for lung resection.

We included randomized clinical trials and observational studies (case-control, case-series, retrospective and prospective cohorts). Systematic, narrative reviews and meta-analysis were used to identify further eligible primary studies through screening of their included studies.

We concluded on the following detailed search strategy:

#1: (“Lung Neoplasms”[Mesh] OR (lung cancer) OR (lung nodule) OR (lung mass) OR (lung carcinoma))
#2: (“Thoracic Surgery”[Mesh] OR “Thoracotomy”[Mesh] OR “Thoracic Surgical Procedures"[Mesh] OR “Thoracic Surgery, Video-Assisted”[Mesh] OR “Robotic assisted thoracic surgery” OR “RATS” OR “VATS”)
#3: (“Indocyanine Green”[Mesh] OR (ICG) OR (Indocyanine Green) OR (near infrared range) OR (NIR)).
#1 AND #2 AND #3

A two-stage screening process was performed until 30th April 2022 against our inclusion and exclusion criteria from the following electronic bibliographic databases: MEDLINE, EMBASE, CENTRAL and Scopus. We also assessed for eligibility the first 300 articles from Google Scholar in order to identify suitable grey literature (15). The databases for MEDLINE and EMBASE were screened together using OVID. Initially, records were screened by title and abstract and then duplicate studies were identified and removed across the different electronical platforms (Ovid, CENTRAL, Scopus and Google Scholar) using EndNote X9. For the second stage of screening we performed full text review of all eligible studies from the title and abstract screening. Both stages were performed by two authors (AG, SL) and any disagreements were resolved through discussion. If a consensus could not be reached between the two authors, then the other members of the team were consulted.

### Data collection

Data extraction forms were designed, reviewed and approved by all authors involved in the project following guidance of Cochrane Handbook for systematic reviews (sample of the form attached at the end of Appendix) (16). The collected data from the eligible studies included:

(1) General Information about the study and its methods: authors, year of publication, name of publishing journal, country of origin of where the study was conducted, type of study, study population inclusion criteria, methods used for analysis of outcome.
(2) Demographics and baseline characteristics of study participants: total number of patients who underwent ICG localization of lung nodules, approach of ICG localization, concentration and volume of administered ICG, technology of NIR intra-operative identification, the patients age, gender, number of nodules, size of nodules, distance of the nodule from the lung surface, final nodule histology.
(3) Procedural Characteristics: Surgical approach used, type of lung excision performed, duration of localization, duration of the lung resection procedure.
(4) Outcome of the study: Intra-operative identification success rate of fluorescent substance, reasons for localization failure, complications from localization, complications after the end of the procedure.

Data were extracted in duplicate by two authors (AG, SL), with disagreements again resolved through discussion and further consultation with the team if required.

### Quality assessment

The quality assessment for risk of bias and applicability of the test was performed according to Quality Assessment of Diagnostic Accuracy Studies 2 (QUADAS 2) (17). Two authors (AG, SL) completed independently their assessment for every eligible study across the four key domains of the QUADAS 2 tool for risk of bias (Low/High/Unclear) and the three key domains of the same tool on applicability. The index test was the localization technique with ICG while the reference standard was the identification of each localized nodule on histology. Any disagreements on the overall score between the two assessors were resolved through discussions with the rest of the team.

### Data and statistical analysis

Details from the included studies are presented with summary tables for study characteristics and quality assessment. Continuous variables are expressed as means and SD or as median and IQR depending on data distribution. Categorical variables are expressed as proportions and percentages.

Prior to data collection, we aimed to perform a meta-analysis on test accuracy studies using hierarchical summary receiver operating characteristic (HSROC) and the bivariate random-effects models (18). Our goal was to plot the summary receiver operating characteristic (SROC) curve which would include the prediction region, the summary point and its confidence region. In cases where the data for one of the localization techniques was not sufficient for that analysis, that was presented with tables with narrative purposes.

For the meta-analysis of the sensitivity and specificity of each localization method we adhered to the following assessments: For CT-guided and endobronchial ICG localization of pulmonary nodules, the administration of ICG was used as a marking dye to guide surgical excision and not as an identification for malignancy. Therefore, true positive (TP) was considered a lesion which was fluorescent after ICG localization and was found completely resected on histopathology. False positive (FP) was considered a lesion which was fluorescent after ICG localization and was either absent or not completely resected on histopathology. True negative (TN) was considered a resected lesion with no identified nodules on histopathology that was not intra-operatively fluorescent because it was not found for localization with ICG. False negative (FN) was considered a lesion which was not fluorescent after ICG localization either due to localization failure or because the fluorescent dye was not identifiable intra-operatively and if resected, there was a nodule present on histopathology. The lesions that were not resected despite pre-operative planning due to wrong localization were considered localization failures and therefore were considered false negative.

Those assessments were different for the intravenous ICG administration because that technique aimed at identifying malignant lung nodules in the lung parenchyma as it relied on previous evidence suggesting that ICG could remain in tumors 24 h after intravenous injection by enhanced permeability and retention (EPR) effect (19). For that reason, we considered true positive (TP) lesions those which were fluorescent intra-operatively while also found to be malignant on histopathology. False positive (FP) was considered a lesion which was fluorescent but was not malignant on histopathology. True negative (TN) was considered a resected lesion that was not fluorescent and was not malignant on histopathology. False negative (FN) was considered a resected lesion which was not fluorescent but was malignant on histopathology and all localization failures that resulted in malignant lesions to remain unresected.

The accuracy of each technique was calculated by the model: (TN + TP)/(TN + TP + FN + FP). Data analysis and synthesis was performed using STATA 17 (StataCorp. 2015. Stata Statistical Software: Release 17. College Station, TX: StataCorp LP).

The results are presented according to PRISMA guidelines.

## Results

**Figure 1:**
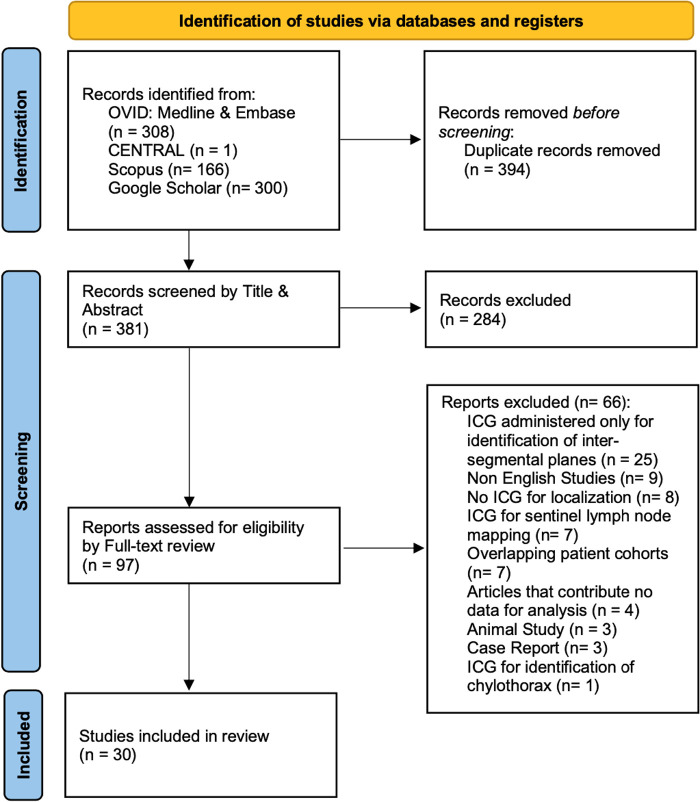
PRISMA flow diagram. ICG: Indocyanine Green.

Our initial search identified 775 studies across the 5 electronic databases. After removal of 394 duplicates, we screened 381 studies by title and abstract. Of the 381 articles, 97 full-text articles were assessed. The second stage of the screening process resulted in 30 studies to be included for further analysis (Figure 1) (19–48).

Overall, there were 1,776 patients who underwent ICG localization of pulmonary nodules in the 30 eligible studies. These studies were subsequently categorized on three subgroups according to the administration technique of ICG for localization. The three techniques which were identified from this systematic review of the literature were CT guided, under bronchoscopy and through peripheral intravenous administration (Table 1). Those included 13, 12 and 8 studies subsequently to each group. In three of the studies, the investigators performed both CT guided and endobronchial localization of the pulmonary nodules (30–32). There was one study that performed both CT guided localization with indigo carmine in all of their patients (*n* = 12) but 3 patients (16 nodules) received ICG IV 24 h pre-operatively and was thus included in our IV group (48). All the studies were conducted in single centers and 63% of them had retrospective study design (19/30). There were four studies that investigated the effectiveness of ICG localization in pediatric population (41, 46–48) while five studies assessed the performance of ICG localization in patients with potential pulmonary metastases (43–47).

**Table 1:** Characteristics of the included studies.

Study	Country	Type of Study	Period	Patients	Localization	NIR System
Hsu et al. (20)	Taipei Veterans General Hospita, Taiwan	Retrospective Single Center	June 2019–November 2020	46	EMN percutaneous (CT)	Olympus Visera Elite II; Olympus, Tokyo, Japan or 1688 AIM 4K platform; Stryker, San Jose, California, USA
Wu et al. (21)	The Fourth Affiliated Hospital of China Medical University, Shenyang, China	Retrospective Single Center	September 2019 – March 2020	32	CT	Not reported
Ding et al. (22)	Peking Union Medical College Hospital, Beijing, China	Retrospective Single Center	October 2020- February 2021	65	CT	Not reported
Li et al. (23)	First Affiliated Hospital of Guangzhou Medical University, Guangzhou, China	Retrospective Single Center	May 2019–May 2020	471	CT	Stryker, Kalamazoo, MI, USA
Zhang et al. (24)	Guangdong General Hospital, Guangzhou, China	Prospective Single Center	January 2018–April 2018	35	CT	Not reported
Li et al. (25)	Tuen Mun Hospital, Hong Kong	Retrospective Single Center	July 2018–July 2019	19 (6 ICG, 13 Hook wire)	CT	Not reported
Chang et al. (26)	Chang Gung Memorial Hospital-Linkou, Taiwan	Retrospective Single Center	July 2017–May 2021	175	CT	PINPOINT (Stryker, Kalamazoo, MI, USA)_or a D-Light (Karl Storz, Tuttlingen, Germany)
Nagai et al. (27)	Osaka National Hospital, Osaka, Japan	Retrospective Single center	March 2007 to June 2016	37	CT	Not reported
Ujiie et al. (28)	Toronto General Hospital, Toronto, Ontario, Canada	prospective phase I clinical trial	May 2014 and March 2016	20	CT + microcoil	Pinpoint System; Novadaq Technologies Inc, Mississauga, Ontario, Canada
Zhong et al. (29)	Gansu Province People’s Hospital, Lanzhou, China	Prospective Single Center	Mach 2016–Aug 2019	30	CT + micro coil	Not reported
Yan-Long Yang at al. (30)	Guangdong Province, Shantou Central Hospital and Lung Research Institute of Guangdong Provincial People’s Hospital, China	Retrospective Single Center	Jan 2018–Dec 2019	47	CT (35)& Bronchocoschopy (12)	Not reported
Yeasul Kim et al. (31)	University Guro Hospital Seoul, Korea	Retrospective Single Center	March 2016–July 2019	31	CT (28) & EMNB (3)	Pinpoint thoracoscope (Novadaq Technologies Inc., Mississauga, ON, Canada) or Firefly fluorescence imaging, da Vinci Si system (Intuitive Surgical, Inc., Sunnyvale, CA) + C-arm fluoroscopy (Koninklijke Philips, N.V., Amsterdam, the Netherlands)
Anayama et al. (32)	Kochi Medical School, Kochi University, Japan	Retrospective Single Center	January 2013 – December 2018	61 (3 groups)	CT (15) & X-ray fluoroscopy-guided bronchoscopy (24) & cone-beam computed tomography augmented fluoroscopy-guided bronchoscopy (22)	Stryker, Kalamazoo, MI, USA
Sekine et al. (33)	Tokyo Women’s Medical University Yachiyo Medical Center, Tokyo, Chiba, Japan	Prospective, single-centre, phase II, feasibility study	December 2017 – July 2020	28	Bronchoscopy	PINPOINT; Stryker, Kalamazoo, MI, USA
Yanagiya et al. (34)	NTT Medical Center, Japan Tokyo	Retrospective Single Center	April 2020 – September 2020	5	Bronchoscopy	VISERA ELITE II system (Olympus, Tokyo, Japan) or Da Vinci Xi system (Intuitive Surgical Inc., Tokyo, Japan)
Zhang et al. (35)	First Affiliated Hospital of Guangzhou Medical University, Guangzhou, China	Retrospective Single Center	October 2018–March 2021	173	EMNB	Stryker, Kalamazoo, MI, USA
Geraci et al. (36)	New York University Langone Health, New York, USA	Retrospective Single Center	January 2010–October 2018	245	EMNB	(Firefly, Intuitive Surgical, Sunnyvale, CA)
Yang et al. (37)	National Taiwan University Hospital, Taipei, Taiwan	Prospective Single Center	July 2018–March 2019	51	computed tomography-derived augmented fuoroscopy guided Bronchoscopy	Pinpoint System; Novadaq Technologies Inc., Mississauga, Ontario, Canada
Abbas et al. (38)	Temple University Hospital, Philadelphia, USA	Retrospective Single Center	May 2013–August 2015	51	EMNB	Firefly Mode; Intuitive Surgical Inc, Sunnyvale, Calif
Ng, Calvin et al. (39)	Sun Yat-sen University Cancer Center, Guangzhou, China	Retrospective Single Center	N/R	6	EMNB	OPAL1, Karl Storz, Germany
Hachey et al. (40)	Boston Medical Center, USA	Prospective pilot trial Single center	March and December 2015	12	Bronchoscopic	PINPOINT® system (Novadaq, Mississauga, Canada)
Harris et al. (41)	Westchester Medical Center, Valhalla, NY, USA	Retrospective Single Center	May 2018–October 2020	8 (Paeditric Population)	Bronchoscopy	Not reported
Yamin Mao et al. (42)	Peking University People’s Hospital, Beijing, China	Prospective clinical trial	August 2015-October 2016	36	IV	SUPEREYE system by Key Laboratory of Molecular Imaging, Chinese Academy of Science and e D-Light P system by Karl Storz
Okusanya et al. (19)	University of Pennsylvania School of Medicine, Philadelphia, Pennsylvania, USA	Prospective pilot clinical trial	January 2012–July 2012	18	IV 24 h pre-operatively	BioVision, Inc., PA
Hamaji et al. (43)	Kyoto University Hospital, Kyoto, Japan	Prospective pilot study singles center	March 2017–March 2018	22 (Metastasis)	IV 12–24 pre-op (3), Intra-operatively (next 14), Intra-operatively (next 14), Higher dose intra-operatively (next 6)	Advanced Imaging Modality, STRYKER), or thoracoscopic near-infrared imaging (PINPOINT Endoscopic Fluorescence Imaging System, NOVADAQ, Japan)
Keating et al. (44)	Perelman School of Medicine, Pennsylvania USA	Prospective Single Center	July 2012–December 2015	8 (Metastasis)	IV 24 h Pre-operatively	Iridium imaging system (Visionsense, New York, NY)
Predina et al. (45)	Perelman School of Medicine, Pennsylvania USA	Prospective Open label Clinical Trial	November 2014–September 2017	30 (Metastasis)	IV 24 h Pre-operatively	Iridium imaging system (Visionsense, New York, NY)
Kitagawa et al. (46)	Kanagawa Children’s Medical Center, Yokohama, Japan	Retrospective Single Center	October 2012–September 2014	10 (Metastasis/ Paediatric Population)	IV	Photodynamic Eye (PDE), Hamamatsu Photonics, Hamamatsu, Japan
Whitlock et al. (47)	Baylor College of Medicine, Houston, Texas, USA	Retrospective Single Center	2016–2020	5 (Paediatric Population)	IV	STORZ Image1™, MEDTRONIC Elevision™, and the STRYKER SPY™
Yamamichi et al. (48)	Osaka Women’s and Children’s Hospita, Japan	Retrospective Single Center	January 2011–April 2020	3 (Paediatirc Population)	CT (Indigo carmine) & IV (ICG)	Karl Storz, Tuttlingen, Germany

CA, California; CT, Computer Tomography; EMN, Electromagnetic Navigation; EMNB, Electromagnetic Navigation Bronchoscopy; ICG, Indocyanine Green; IV, Intravenous; MI, Michigan; N/R, Not Reported; NY, New York; ON, Ontario; USA, United States of America.

Apart from three pediatric studies, that performed lung resections *via* thoracotomy, the vast majority of papers showed a preference towards VATS or RATS. There was only one adult trial in which the operating team performed their lung resections *via* thoracotomy (19). The vast majority of lung resections was undertaken with sub-lobar excisions in 96% of the operations (1442/1500). Further details of each study's population are illustrated on Table 2.

**Table 2:** Patient and Operative details.

Study	Localization	Patients	Gender	Age, median (IQR)	Number of Nodules	Size of nodules, median (IQR)	Distance of the nodule from the pleura, median (IQR)		Surgical Approach	Lung Resection
Hsu et al. (20)	EMN percutaneous CT	46	20 M, 26 F	N/R	50	9.0 mm (7.4–12.2)	8.2 mm (2.7–13.2)		Uniportal VATS	Segmentectomy: 2 Wedge: 44
Wu et al. (21)	CT	32	15M, 17 F	59.2 years (43–79)	36	0.76 cm (0.4–1.5 )	N/R		Uniportal VATS	Lobectomy: 9 Wedge: 23
Ding et al. (22)	CT	65	16M, 49F	51.3 ± 11.6 years	85	6.3 ± 2.4 mm	9.2 ± 10.0 mm		VATS	N/R
Li et al. (23)	CT	471	196M, 275F	50.8 years (21–84)	512	9.1 mm (6–20)	8.9 mm (1–30)		VATS	Segmentectomy: 80 Wedge: 359 Wedge + segmentectomy: 23 Wedge + lobectomy: 44 Segmentectomy + lobectomy: 6
Zhang et al. (24)	CT	35	9M, 26F	54.7 years (40–79)	35	7 mm (3–20)	8.2 mm (1.1–38.1)		VATS	All Wedge
Li et al. (25)	CT	19 (6 ICG, 13 Hook wire)	8M/11F	61 years (57–65.5)	19	17 mm (10.5–19.5)	N/R		VATS	N/R
Chang et al. (26)	CT	175	78M, 97F	58.76 ± 10.92 years	175	8.34 ± 3.64 mm	5.30 ± 4.53 mm		VATS	Segmentectomy: 71 wedge: 104
Nagai et al. (27)	CT	37	14M, 23F	63.1 years (10–82)	37	9.1 mm (2–22)	9.9 mm (0–33)		N/R	Partial Resection: 34 Segmentectomy: 1 Lobectomy: 2
Ujiie et al. (28)	CT + microcoil	20	10F, 10M	69.5 years (54–82)	20	1.2 cm (0.5–2.4)	1.4 cm (0.2–4.8)		VATS	All Wedge
Zhong et al. (29)	CT + micro coil	30	20M, 10F	62 years (32–75)	42	1.3 cm (0.6–1.9)	1.7 cm (0.5–3.8)		VATS	All Wedge
Yan-Long Yang at al. (30)	CT (35) & Bronchocoschopy (12)	47	CT: 26M, 9F, **Bronchoscopy**: 8M, 4F	CT: 55 years (40–79) **Bronchoscopy** 56 years (34–68)	CT: 35 **Bronchoscopy** 15	CT: 7mm (3–20) **Bronchoscopy** 11 mm (7–18)	CT : 8.2 mm (1.1–38.1) **Bronchoscopy** 12.5 mm (2.4–34.0)		VATS	CT: Wedge: 34 Lobectomy: 1 **Bronchoscopy** Wedge: 12
Yeasul Kim et al. (31)	CT (28) & EMNB (3)	31	17M, 14 F	63.2 ± 9.8 years	31	1.2 cm (0.3–2.5)		16.4 mm (1.0–42.0)	VATS: 22 RATS: 9	All Segmentectomy
Anayama et al. (32)	**Group A**: CT (15) **Group B**: X-ray fluoroscopy-guided bronchoscopy (24) **Group C**: cone-beam computed tomography augmented fluoroscopy-guided bronchoscopy (22)	61 (3 groups)	**Group A**: 10M, 5F **Group B**: 15M, 10F **Group C**: 15M, 7F	**Group A**: Mean Age: 65.0 ± 12.6 years **Group B**: Mean Age: 66.0 ± 9.6 years **Group C**: Mean Age: 67.0 ± 10.6 years	73 **Group A**: 16 **Group B**: 30 **Group C**: 27	**Group A**: 10 ± 3.4 mm, **Group B**: 9.2 ± 3.6 mm, **Group C**: 8.0 ± 4.5 mm		**Group A**: 9.9 ± 7.7 cm: **Group B**: 9.8 ± 8.1 mm: **Group C**: 3.0 ± 6.0 mm	VATS	All Wedge
Sekine et al. (33)	Bronchoscopy	28	17M, 11F	69.4 years (41–83)	28	12.4 ± 4.3 mm		7.2 mm ( 0–20)	VATS	All Wedge
Yanagiya et al. (34)	Bronchoscopy	5	4M, 1F	64 years (62–69)	8 (20 markings)	10 mm (6.5–11.25)		7.5 mm (4–10.25)	VATS: 7 RATS: 1	Wedge: 7 Wedge + Lobeectomy: 1
Zhang et al. (35)	EMNB	173	63M, 110F	52.77 ± 11.08 years	180	9.21 ± 4.81 mm		33.8 ± 10.57 mm	VATS	Wedge: 171 Segmentectomy: 7 Lobectomy: 3
Geraci et al. (36)	EMNB	93	116M, 129F	68 years (18–87)	93	1.7 cm (0.6–4.6)		N/R	RATS	Segmentectomy
Yang et al. (37)	computed tomography-derived augmented fuoroscopy guided Bronchoscopy	51	20M, 31F	56 years (50–63)	61	8.6 mm (7.0–11.8)		15.4 mm (10.6–23.1)	VATS	Wedge: 44 Segmentectomy: 8 Lobectomy: 5
Abbas et al. (38)	EMNB	51	26M, 25F	62.6 years (41-86)	54	13.3 mm (4–44)		22 mm (4–38)	VATS: 2, RATS: 47Thoracotomy: 2	Wedge: 10 Segmentectomy: 13 Lobectomy: 28
Ng, Calvin et al. (39)	EMNB	6	N/R	N/R	6	(2–12 mm)		N/R	VATS	N/R
Hachey et al. (40)	Bronchoscopic	12	2M, 10F	62 ± 6.7 years	14	(0.4–2.2 cm)		(0.1–3.0 cm)	VATS	Wedge: 11 Lobectomy: 1
Harris et al. (41)	Bronchoscopy	8 (Paeditric Population)	7M, 1F	13.4 years	20	(3–24 mm)		(1–15 mm)	VATS	All Wedge
Yamin Mao et al. (42)	Peripheral IV	36	19M, 17F	56 ± 14 years	76	1.4 ± 1.2 cm		N/R	N/R	Wedge: 14 Segmentectomy: 7 Lobectomy: 14 Pneumonectomy: 1
Okusanya et al. (19)	Peripheral IV	18	N/R	60 years (29–78)	23	2.8 cm (0.8–11)		0.4 cm (0–1.3)	Thoracotomy	Wedge: Numbers N/R Segmentectomy: Numbers N/R Lobectomy: Numbers N/R
Hamaji et al. (43)	Peripheral IV	22 (Metastasis)	13M, 9F	67 years (15–82)	22	1.15 cm (0.5–3.5)		0.65 cm (0.01–1.26)	N/R	Wedge: 16 Segmentectomy: 1 Lobectomy: 4 Resection of Pleural lesion: 1
Keating et al. (44)	Peripheral IV	8 (Metastasis)	N/R	N/R	11	1.75 ± 1.4 cm		within 2 cm	VATS	N/R
Predina et al. (45)	Peripheral IV	30 (Metastasis)	16M, 14F	51.5 years (23–79)	61 pre-op 86 intra-op	1.6 cm (0.5–3.2)		(1.3–2.1) cm	VATS: 20 Thoracotomy: 10	All Wedge
Kitagawa et al. (46)	Peripheral IV	10 (Metastasis/ Paediatric Population)	6M, 4F	2 years (1–4.25)	255	Smallest lesion: 0.062 mm		N/R	Thoracotomy	All Wedge
Whitlock et al. (47)	Peripheral IV	5 (Paediatric Population)	N/R	48 months (41–72)	44	Smallest measured lesion was 1.7 mm		N/R	VATS: 5 Thoracotomies: 4	N/R
Yamamichi et al. (48)	Peripheral IV	3 (Paediatirc Population)	2M, 1F	3 years (1–9)	16	3 mm (1–8)		3 mm (1–11)	Thoracotomy	N/R

CT, Computer Tomography; EMN, Electromagnetic Navigation; EMNB, Electromagnetic Navigation Bronchoscopy; F, Female; ICG, Indocyanine Green; IQR, Interquartile Range; IV, Intravenous; M, Male; N/R, Not Reported; RATS, Robotic-assisted thoracoscopic surgery; VATS, Video-assisted thoracoscopic surgery.

### Quality assessment

The quality assessment of the studies demonstrated not only low risk of bias (RoB) on reference standard and flow and timing but also low concern regarding applicability in more than 80% of the included studies (26/30, 28/30 and 28/30 respectively). The index test assessment showed high risk of bias in 36.7% (11/30) which also reflected on the high level of concern regarding applicability in 30% of the studies (9/30). This was the result of studies administering a mixture of ICG with other localization techniques such as micro-coils and other dyes (Iopamidol, Methylene Blue, Indigo Carmine) which introduced significant bias on the assessment of the effectiveness of ICG as independent marker (25, 28, 29, 34, 38, 39, 41). Furthermore, two studies that investigated the IV administration of ICG, had significant variation on ICG dose and time interval between ICG administration and the operation among the participants of each study (43, 47).

**Figure 2:**
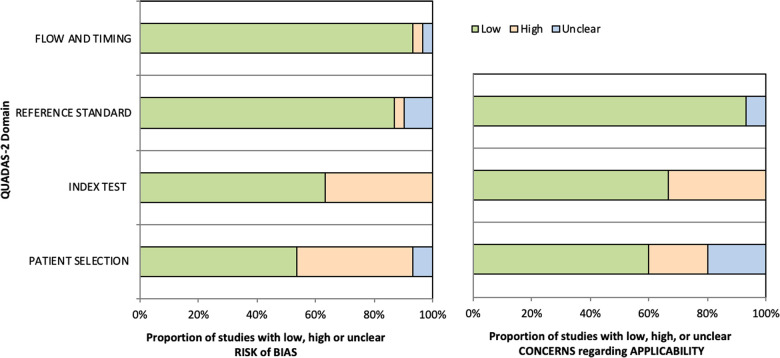
QUADAS-2 tool assessment on Risk of Bias (Left bar chart) and Applicability (Right bar chart).

Finally, the RoB was low in 53% of the studies (16/30) on patient selection. This was predominantly the result of poor population description with regards to co-morbidities and peri-operative data which can be attributed to restrictions which are usually encountered in retrospective studies. The summary of QUADAS-2 assessment is illustrated in Figure 2 and the review of each study can be found in the Appendix.

### Outcome assessment

The primary outcome of this study was to evaluate the success rate for ICG localization. The studies that evaluated the CT-guided and endobronchial techniques did not report any true negative or false positive lesions based on our predefined criteria. Subsequently, this resulted in inability to use the bivariate model or HSROC. We therefore, summarize the findings of our systematic review for these two localization techniques on Tables 3, 4.

**Table 3:** Operative details and outcomes from studies that used CT-guided Indocyanine Green localization.

Study	Patients	ICG dose	Other localization technique	Duration of localization, min, median [range]	Timing between localization and Lung Resection	Duration of lung resection, min, median [IQR]	Total time, min, median (IQR)	Radiation Exposure	Successful Localization	Reasons for Localization Failure	Complications	Histology
Hsu et al. (20)	46	0.3 ml of 0.125 mg/mL	No	10.0 (7.3–15.0)	N/R	55 (40–75)	163 (135–190)	N/R	45/46 (90%)	N/R	3 Persistent a/l >3 d (6.5%), 1 chylothorax (2%)	Invasive Adenocarcinoma: 7, Metastases: 8, Adenocarcinoma *in situ*: 17, Minimally invasive adenocarcinoma: 13 Benign: 13
Wu et al. (21)	32	0.3–1 ml of 2.5 mg/ml	No	8.3 (6–15)	40–60 min	45.3 (38–62)for Wedge & 65 (50–120) for lobectomy	N/R	N/R	33/36 (91.7%)	Diffuse thoracic images: 2, Images not developed: 1	3 dyspnoea & chest pain post-localization (9.4%), 1 post-op AF (reverted) (3%)	All R0 36/36, 28 malignant, 8 Benign, Non-small cell lung cancer: 9, Carcinoma *in situ*: 5, Atypical hyperplasia: 12, Organizing pneumonia: 5, Metastases: 2, Lymphoid tissue: 2
Ding et al. (22)	65	0.03 ml of 25 mg of ICG mixed with 50 ml of Iopamiro	Iopamiro (Iopamidol)	24.52 ± 3.43	From localization to VATS within 60 min	N/R	N/R	N/R	85/85 (100%)	N/A	18 pneumothorax (27.7%), 5 lung parenchymal hemorrhage (7.7%) (no drain required)	All R0, 64 Malignant (75%), 12 Benign, Adenocarcinoma *in situ*: 22, Minimally invasive adenocarcinoma: 23, Invasive adenocarcinoma: 19, Hamartoma: 1, Benign: 12, Granuloma: 1, atypical adenomatous hyperplasia primary (*n* = 5)
Li et al. (23)	471	0.4 ml of ICG (2.5 mg/mL) + 0.8 ml N/S	No	18.0 (10–47)	within 24 h	N/R	N/R	Average Radiation: 281.5 mGy	415/512 (81.1%)	Diffusion of ICG in the thorax	32 pulmonary hemorrhage (6.8%), 28 pneumothorax (5.9%), 2 hemoptysis (0.4%), 2 pleural reaction (0.4%) (no drain required)	**All R0**, Benign: 49, Atypical adenomatous hyperplasia: 26, Adenocarcinoma *in situ*: 28, Minimally invasive adenocarcinoma: 289, Invasive adenocarcinoma: 99, Small cell lung cancer: 1
Zhang et al. (24)	35	0.1–0.2 ml of 2.5 mg/ml	No	28 (18–40)	After satisfactory injection of ICG, patients were sent directly to the operating room	Median 25 (16–30)	N/R	N/R	32/35 (91.4%)	1 insufficient ICG injection, 1 ICG leakage to the thoracic cavity, 1 deep lesion in the lung parenchyma requiring lobectomy	None	33 adenocarcinomas (94.2%), 1 benign disease (2.9%), and 1 metastatic lung cancer from colon cancer (2.9%)
Li et al. (25)	19 (6 ICG/lipoidol mixture only, 13 ICG/lipoidol mixture + Hook wire)	0.2–0.4 ml mixture of ICG with lipiodol in 1 to 9 or 2 to 8 ratio	ICG/lipoidol mixture + spiral end hookwire (SOMATEXR Lung Marker System®, Somatex Medical Technologies GmbH, Germany)	Average 42.6	5 hrs after the localization	Average 73.4 min	N/R	N/R	19/19 (100%)	N/A	All developed trace to mild <5% pneumothorax (no drain required), Some patients experienced wound pain and mild cough	lung adenocarcinomas (*n* = 13); minimal invasive adenocarcinoma (*n* = 1); atypical adenomatous hyperplasia (*n* = 1); adenocarcinoma in-situ (*n* = 1); interstitial fibrosis/scar (*n* = 3).
Chang et al. (26)	175	0.3 ml of 2.5 mg/ml	No	14.71 ± 6.02	13.67 ± 7.47 min	N/R	N/R	N/R	172/175 (98.3%)	1 ICG spealage. In the 3 cases that ICG was not visible there was erroneous injection of the ICG dye into the deep lung parenchyma	6 small pneumothorax (3.4%) (no drain required), 1 persistent air leak >5 days (0.6%), 1 empyema (0.6%)	Malignant 111 (63.4%), Benign 64 (36.6%)
Nagai et al. (27)	37	0.5 ml deployed into the lung parenchyma adjacent to the nodule, and then another 0.5 ml was injected while withdrawing the needle; thus, in total, 1 ml (12.5 mg/ml)	No	19.4 min (12–41)	102 min (IQR: 84–179).	N/R	N/R	N/R	35/37 (94.6%)	2 localization failures occurred owing to dye spreading and severe pleural adhesion	3 mild pneumothorax (8.1%) (no drain required), 5 Cough (13.5%), 1 Mild Haemoptysis (2.7%)	All R0, 31 nodules were malignant (Adenocarcinoma: 20, Metastases: 11) and 6 were Benign
Ujiie et al. (28)	20	100 to 150 μl of 0.125 mg/ml	Vortex microcoil	35 (19–59)	Immediately after localization	54 (28–84)	N/R	N/R	18/20 with ICG (90%) (2/20 detected the microcoils with fluroscopy)	1 Failed ICG case: nodule 1.0 cm in size was located 4.8 cm from the pleura, 1 failed ICG case due to unsuccessful lung deflation	None	**All R0**, Adenocarcinoma: 16, SCC: 1, Small Cell: 1, Focal perivascular lymphoid infiltrate: 1, Metastases: 1
Zhong et al. (29)	30	2.5 mg/ml using 25% human serum albumin with 100 to 150 ml of ICG	microcoil	Median 25 (19–49)	Immedately aftter microcoil position	50 (42–80)	N/R	N/R	42/42 (100%)	N/A	None	All R0 21 adenocarcinomas, 3 squamous cell carcinomas, 3 adenocarcinoma squamous cell carcinomas and 3 small cell carcinomas
Yan-Long Yang at al. (30)	35	ICG/iopamidol mixture 0.3–0.5 ml of ICG concentration 0.125 mg/ml	ICG/iopamidol mixture	within 90 minutes	N/R	N/R	N/R	N/R	33/35 (94.3%)	N/R	5 pneumothorax (14%) - no chest drain required	All R0 , atypical adenomatous hyperplasia: 1 adenocarcinoma *in situ*: 12, microinvasive adenocarcinoma: 7, infiltrating adenocarcinoma: 13, Metastases: 1, Benign: 1
Yeasul Kim et al. (31)	28	0.3 ml of 0.5 mg/ml emulsion of 10% ICG and 90% lipiodol	lipiodol mixture with ICG	14.3 ± 3.1 min	310.1 min (118–1882)	168.7 ± 53.3 min	N/R	N/R	28/28 (100%)	N/A	1 mild pneumothorax (3.2%) (no drain required) Post-op: 5 AF 16.1%), 2 pneumonia (6.4%), 3 air leak (9.7%)	100% Malignant 28 had adenocarcinoma and 3 squamous cell carcinoma
Anayama et al. (32)	15	50–100 µl of ICG/iopamidol marking solution. Diluting ICG (2.5 mg/ml, 10 ml) 100-fold with iopamidol (Iopamiron 370). 1 ml Syringe was filled	ICG/iopamidol mixture	N/R	localization was performed on the day of the operation	N/R	N/R	N/R	15/16 (93.8%)	1 failed because of the development of a small secondary pneumothorax that occurred after the first VATS marking	3 Small pneumothorax (20%) (no drain required)	N/R

AF, Atrial fibrillation; a/l, air leak; CT, Computer Tomography; ICG, Indocyanine Green; IQR, Interquartile Range; mGy, milligray; N/A, Not Applicable; N/R, Not Reported; N/S, Normal Saline; SCC, Squamous Cell Carcinoma; VATS, Video-assisted thoracoscopic surgery.

**Table 4:** Operative details and outcomes from studies that used bronchoscopy Indocyanine Green localization.

Study	Patients	ICG dose	Other localization technique	Duration of localization, Median (IQR)	Timing between localization and Lung Resection, Median (IQR)	Duration of lung resection, Median (IQR)	Radiation Exposure	Successful Localization	Reasons for Localization Failure	Complications	Histology
Sekine et al. (33)	28	0.25 mg/ml of ICG was further diluted in 70 ml of saline and 20 ml of autologous blood for a 10-fold dilution of ICG. 10 ml ICG in each targeted 4th–6th bronchial branch followed by 200-400 ml of air.	None	5–15 min	15–30 min.	69.9 min (33–116)	N/R	28/28 (100%)	N/A	2 (7.1%)prolonged a/l (>5 d) requiring pleurodesis	**All R0** Invasive adenocarcinoma: 7 Minimally invasive adenocarcinoma: 5 Adenocarcinoma *in situ*: 4 Neuroendocrine carcinoma: 1 Metastases: 11
Yanagiya et al. (34)	5	0.1 ml ICG + and 1.0 ml indigo carmine followed by 20 ml of air	Indigo Carmine	15 min (13–17)	N/R	N/R	N/R	19/20 (95%)	5 Marking failure from IC and 1 from ICG but no justification	None	**All R0** Adenocarcinoma *in situ*: 4 Benign: 2 Metastases: 2
Zhang et al. (35)	173	0.3 ml ICG (0.6 mg/ml) followed by 2 ml air	None	for 1 target: 9.75 ± 6.5 min for 2 targets: 11.35 ± 5.27 min for 3 targets: 24.54 ± 17.05 min	Localization performed in the theatre and then immediately proceeded to VATS	N/R	N/R	178/181 (98.3%)	N/R	None	**All R0.** Benign Lesions: 35 Atypical adenomatous hyperplasia: 10 Adenocarcinoma *in situ*: 17 Minimally invasive adenocarcinoma: 82 invasive adenocarcinoma: 34 Metastases: 2
Geraci et al. (36)	93	10 ml of sterile water in a 25-mg bottled powder of ICG Peritumoral injection of 0.5 ml intra-brochial.	Remaining 9.5 ml given IV peripherally for inter-segmental plane	9 min (3–31)	Localization performed in the theatre and then immediately proceeded to RATS	86 min (43–250)	N/R	80/93 (86%)	inaccurate injection of indocyanine green from the target lesion (7), failure of the navigational bronchoscopy system or software (3), and pleural perforation (3)	31 (12.6%) prolonged a/l >24 h after surgery, 10 (4%)AF, 9 (3.6%) urinary retention, 2 (2.2%)Pneumonia 1 (1%) Stroke	Benign: 42 Adenocarcinoma: 106 SCC: 54 Neuroendocrine tumor: 12 Carcinosarcoma of lung: 2 Metastases: 28
Yang et al. (37)	51	1–2 ml of 0.25 mg/mL	17 pts (33.3%) Indigo carmine (20 mg/ml), 28 contrast diluted dye (54.9%)	28 min (23–34)	16.4 h (4.2–20.7)	N/R	Median radiation dose of a single DynaCT scan: 1592.9 µGym2 Median radiation dose of fuoroscopy: 405.1 µGym3	34/34 (100%)	N/A	None	Primary lung adenocarcinoma: 40 Lung metastases: 8 Benign: 13
Abbas et al. (38)	30	1–2 ml injected bronchoscopically	Methylene Blue in 21 pts mixture of Isovue, Methylene Blue, and ICG 30 pts.	29.1 min (12–45)	Immediately after EMNB	212 min (140–290)	N/R	29/30 (96.7%)	N/A	2 (3.9%) AF, 2 (3.9%)AKI, 1 (2%) recurrent pneumothorax (requiring drain), 1 (2%) re-intubation for hypoventilation	Benign: 11 Adenocarcinoma: 32 SCC: 2 Neuroendocrine: 2 Metastases: 7
Ng, Calvin et al. (39)	6	0.2 and 0.5 ml of triple-contrast dye mixture of equal volumes of iohexol imaging contrast (Omnipaque, GE Healthcare, Chicago, Illinois, United States), methylene blue, and ICG	Iohexol imaging contrast + Methylene Blue	N/R	N/R	N/R	N/R	N/R	N/R	N/R	N/R
Hachey et al. (40)	12	0.5–1 ml of ICG (2.5 mg/ml) diluted to 25% human serum albumin.	None	Average: 34.5 min	65 ± 31 min	N/R	N/R	14/14 (100%)	N/A	None	**All R0** 14/14 malignant Adenocarcinoma: 9 SCC: 1 Metastases: 2
Harris et al. (41)	8 (Paeditric Population)	methylene blue dye and indocyanine green dye. 0.5 ml of either dye is injected for each tattoo	Methylene Blue	<30 min	N/R	N/R	N/R	20/20 (100%)	N/A	None	Benign: 8 Metastastes:2
Yan-Long Yang at al.	12	ICG/iopamidol 0.3–0.5 ml, 0.125 mg/ml	N/R	N/R	N/R	N/R	N/R	15/15 (100%)	N/A	None	**All R0** Atypical adenomatous hyperplasia: 1 adenocarcinoma *in situ*: 3 Microinvasive adenocarcinoma: 3 Infiltrating adenocarcinoma: 4 Metastases: 0 Benign: 4
Yeasul Kim et al.	3	0.3 ml of 0.5 mg/ml emulsion of 10% ICG & 90% lipiodol	lipiodol mixture with ICG	25 ± 4.1 min	310.1 min(118–1882)	168.7 ± 53.3 min	N/R	2/3 (66.7%)	1 patient had positive ressection margins	5 (16.1%) AF, 2 (6.4%)pneumonia, 3 (9.7%) air leak	100% Malignant Adenocarcinoma: 28 SCC: 3
Anayama et al.	46	50–100 µl of ICG/iopamidol marking solution. Diluting ICG (2.5 mg/ml, 10 ml) 100-fold with iopamidol (Iopamiron 370). 1 ml Syringe was filled	ICG/iopamidol mixture	N/R	**Group B:**Localization was performed the day before the operation **Group C:**localization performed in hybrid theatre	N/R	N/R	**Group B:** 28/30 (93.3%) **Group C**: 25/27 (92.6%) **Overall**: 53/57 (93%)	**Group C**: In the 2 unsuccessful cases, the marker was placed deeper into the lesion in the lung parenchyma.	None	N/R

AF, Atrial fibrillation; AKI, Acute Kidney Injury; a/l, air leak; CT, Computer Tomography; EMNB, Electromagnetic Navigation Bronchoscopy; IC, Indigo Carmine; ICG, Indocyanine Green; IQR, Interquartile Range; IV, Intravenous; mGy, milligray; N/A, Not Applicable; N/R, Not Reported; RATS, Robotic-assisted thoracoscopic surgery; SCC, Squamous Cell Carcinoma; VATS, Video-assisted thoracoscopic surgery.

The ICG localization of pulmonary nodules under CT guidance had an overall success rate of 97.6%, with median success rate 94.3% (IQR: 91.4%–100%). The accuracy of the technique was estimated at 97.3%. There were only 2.4% (26/1089) of false negative ICG markings which resulted from localization failure, most commonly due to ICG spillage and subsequent diffused dye in the chest cavity. There were 15.2% (150/989) of patients who developed complications during their hospitalization. In 131 of these patients their complication was related to CT guided localization. However, in 120 of them the complications were small traces of pneumothorax and intra-parenchymal hemorrhage which was only visible on CT during guidance. None of these patients required a chest drain or any other intervention for these complications. The other 11 patients experienced either dyspnea, chest pain or mild hemoptysis after localization. There were no reported allergic reactions in any of the studies. The duration of localization time was reported by 12 studies and ranged from 6 to 59 min while the lung resection time was reported by 7 studies with a range from 16 to 221 min. From the 11 studies that reported the timing between localization and lung resection, the vast majority of them (90.9%, 10/11) proceeded with lung resection within a few hours.

From the 12 studies that ICG localization of the pulmonary nodules was performed under bronchoscopy had an overall success of 95.4%, median 98.3% (IQR: 94%–100%). The accuracy of bronchoscopy-guided ICG localization was calculated at 95.5%. The overall false negative ICG markings were 4.4% (20/458). Only in 1 study, the patients underwent bronchoscopic localization of their nodules the day before their scheduled lung resection (32). That was only offered to 24 patients of that study during an interval in which the department was setting up a hybrid thoracic theatre. There were no reported complications following ICG localization. The overall duration of endobronchial localization was reported by 75% (9/12) studies and had a reported range from 3 to 45 min. One study showed that localization time increased significantly when multiple nodules were localized and that was estimated at 9.75 ± 6.5 min, 11.35 ± 5.27 min and 24.54 ± 17.05 min for 1, 2 and 3 targets respectively (35).

**Figure 3:**
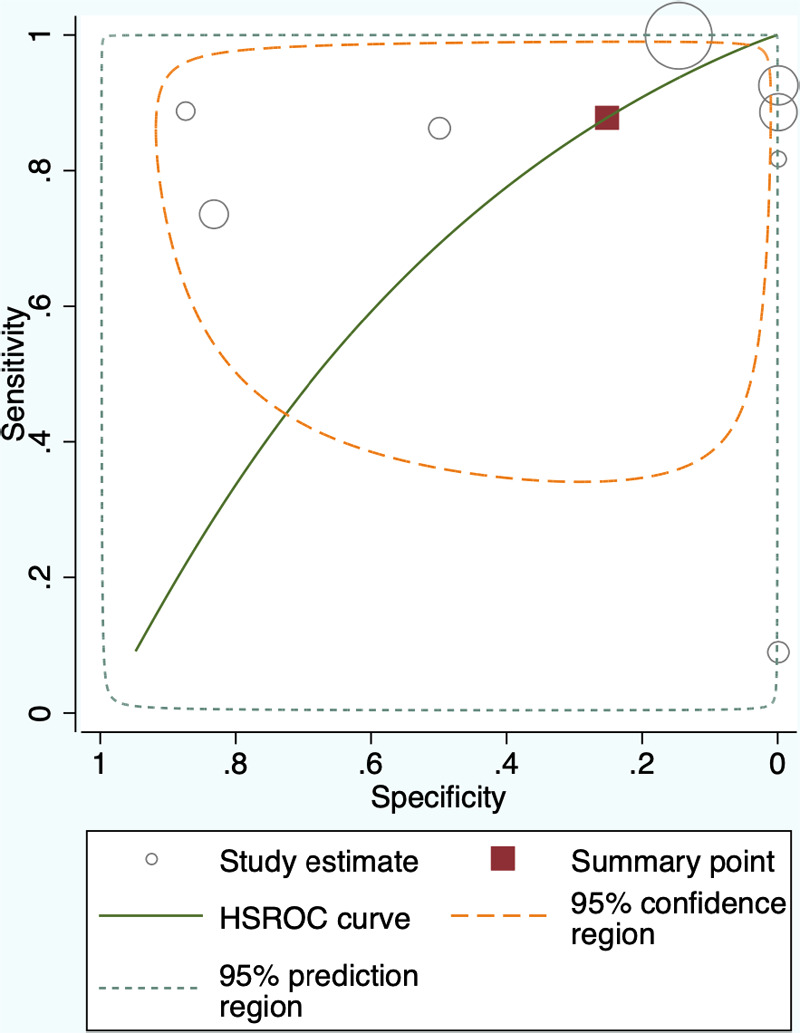
Summary Receiver Operating Characteristic (SROC) curve for Intravenous administration of Indocyanine Green for lung cancer localization. HSROC: hierarchical summary receiver operating characteristic.

The 8 studies that investigated the effectiveness of ICG localization after peripheral IV administration reported enough data that allowed for bivariate model and HSROC to function (Tables 5, 6). The point estimates for Sensitivity and Specificity were 88% (95% CI: 59%–0.97%) and 25% (95% CI: 0.04%–0.74%) respectively (Figure 3). These results are illustrated in detail on the SROC curve and Table 6. The accuracy of IV ICG administration for malignant lung tumor localization was estimated at 83.5%. There were no ICG localization related complications in any of the patients. The time interval between ICG administration and the lung resection ranged from 12 h to 96 h but the dose was mostly unanimously administered at 5 mg/kg in 87.5% of the studies (7/8). The only studies that administered a smaller dose was a pediatric study and an adult study that later raised the dose to 5 mg/kg after poor localization success (43, 48).

**Table 5:** Operative details and outcomes from studies that used intravenous Indocyanine Green localization.

Study	Patients	ICG dose	Other localization technique	Timing between ICG administartion and Lung Resection	Duration of lung resection	Radiation Exposure	Successful Localization	Reasons for Localization Failure	Complications	Histology
Yamin Mao et al. (42)	36	ICG 5 mg/kg	None	24 h pre-operatively	N/R	N/A	68/76 (89.5%)	The other 8 were 1.3 cm or more beneath the pleural surface.	None	71 malignant and 5 benign Adenocarcinoma: 30 SCC: 4 Benign: 5, Metastases: 34 atypical adenomatous hyperplasia: 3
Okusanya et al. (19)	18	5 mg/kg ICG	None	24 h pre-operatively	N/R	N/A	19/23 (82.6%) nodules were fluorescent in the patient, 2 were fluorescent after dissection, 2 were not fluorescent at all.	From the non-fluorescent nodules 1 was Chronic pulmonary emboli and 1 was melanoma metastasis	None	Adenocarcinoma: 10 SCC: 5 Metastases: 4 Adenosquamous: 1 Typical carcinoid: 1
Hamaji et al. (43)	22 (Metastasis)	0.25 mg/kg of ICG in 17 first patients 0.5 mg/kg of ICG in the last 5 patients	None	12–24 h pre-operatively (4 patients), Intra-operatively (next 18 patients)	N/R	N/R	2/22 (9%)	N/R	None	Metastases: 20 SCC: 1 Adenocarcinoma:1
Keating et al. (44)	8 (Metastasis)	5 mg/kg of intravenous ICG	None	24 h pre-operatively	N/R	N/A	9/11 (81.8%)	Those 2 nodules were >1 cm from the pleura surface but were fluoresent after ex-vivo incision of the excised specimen	None	All R0 melanoma (*n* = 4), osteogenic sarcoma (*n* = 2), renal cell carcinoma (*n* = 2), chondrosarcoma (*n* = 1), leiomyosarcoma (*n* = 1), colorectal carcinoma (*n* = 1)
Predina et al. (45)	30 (Metastasis)	intravenous ICG (5 mg/kg)	None	24 h pre-operatively	N/R	N/A	76/82 (92.7%) of malignancies detected + 3 false positives which were Benign.	non-fluorescent metastases were deeper than fluorescent metastases (2.1 cm vs 1.3 cm; *p* = 0.03)	None	Sarcomas: 82 benign lymphoid aggregates: 3
Kitagawa et al. (46)	10 (Metastasis/ Paediatric Population)	0.5 mg/kg of ICG	None	24 h pre-operatively	N/R	N/A	221/221 (100%) of malignancies were dtected + 34 false positives which were benign + 5 True negatives which were Benign	N/A	None	Hepatoblastoma: 221 Benign: 34
Whitlock et al. (47)	5 (Paediatric Population)	0.2—0.75 mg/kg	None	24–96 h pre-operatively	N/R	N/A	28/44 (63.6%) were ICG +, 5 were True−and 1 was were False + which Benign **28/38 (73.7%)** malignant nodules were succesfully localized	10 False negative nodules were malignant.	None	Malignant: 38 Benign: 6
Yamamichi et al. (48)	3 (Paediatirc Population)	0.5 mg/ kg ICG.	CT guided localisation with 0.5–1.0 ml of indigo carmine dye	24 h pre-operatively	N/R	N/A	8/16 (50%) of all resected nodules were ICG+ **8/9 (88.9%)** malignant nodules detected	True+: 8 True −: 7 False +: 0 False −: 1	2 intra-operative pneumothorax after localisation, 2 atelectasis	Malignant: 9

CT, Computer Tomography; ICG, Indocyanine Green; IQR, Interquartile Range; mGy, milligray; N/A, Not applicable; N/R, Not Reported; SCC, Squamous Cell Carcinoma.

**Table 6:** Summary statistics for intravenous Indocyanine Green localization.

Summary Point	Coef	St Error	Lower 95%CI	Higher 95%CI
Sensitivity	0.88	0.88	0.59	0.97
Specificity	0.25	0.21	0.04	0.74
DOR	2.42	3.22	0.18	32.74
LR+	1.17	0.34	0.66	2.06
LR-	0.48	0.51	0.06	3.86
1/LR-	2.06	2.18	0.26	16.43

CI, Confidence Interval; Coef, Coefficient; DOR, Diagnostic Odds Ratio; St Error, Standard Error; +LR, Positive likelihood ratio; -LR, Negative likelihood ratio.

## Discussion

Following the 10-year follow-up results from the NELSON trial, the low-dose CT lung cancer screening showed reduced lung-cancer mortality among high-risk population and higher detection of earlier stage lung cancer (4). This finding in combination with the recent publications of the VIOLET trial and the study by the Japan Clinical Oncology Group/West Japan Oncology Group (JCOG/WJOG4607L) show a clear trend towards minimally-invasive techniques and anatomical sublobar resections for smaller lung cancers (5, 6). Therefore, localization techniques that will guide the operating surgeon to safely identify intra-operatively constantly smaller lung cancers, show an increased scientific interest over the last decade. In this study, we performed a thorough systematic review of the literature and meta-analysis on ICG localization techniques for detection of lung malignancies.

There have been several localization techniques already described in the literature for lung cancer. Hook-wire localization was one of the first described and most commonly used techniques which has also been widely performed in surgical oncology for other organs such as breast (49, 50). However, hook-wire insertion for lung cancer carries a relatively high rate of complications such as pneumothorax and wire migration or dislodgement (51). Furthermore, some studies have reported cases of massive air embolism following hook-wire insertion which is life-threatening complication (52, 53).

Micro-coil localization is another reported approach with documented high success rate (96.9%–100%) (54, 55). Their use for lung nodule localization in thoracoscopic surgery has been performed since 1994 (56). Micro-coils overcome some of the hook-wire complications as they are being deployed in the lung parenchyma without parts of wire being left outside the chest. Migration of the micro-coil has been described and can lead to procedure failure albeit less frequently compared to hook-wire (57). One of the main disadvantages in this technique is that it requires hybrid operating theatre as the lung resection has to be performed under fluoroscopy guidance in order to safely resect the targeted nodule. This demands adequately trained theatre staff and the team needs to wear lead-protecting equipment, to counteract the radiation exposure from fluoroscopy, which adds weight on the back of operating surgeons and could restrict dexterity and range of movement.

In attempt to resolve migration of metallic markers, researchers have focused on investigating multiple dyes and contrast agents that can identify lung nodules. These are Methylene Blue (MB), Indigo Carmine (IC), Barium, Lipiodol and Iopamidol (57). The latter three dyes, despite their successful intra-operative localization rate (93.3%–100%) require fluoroscopic guidance during lung resection. Furthermore, Barium localization induces some acute inflammatory response and edema on the surrounding lung parenchyma which can impair the histopathological diagnosis (55). MB, is a widely used dye for localization of peripheral lung nodules both under CT guidance and under bronchoscopy with good success rates (93.3%–100%) (57, 58). Even though it has shown satisfactory outcome, a significant restriction in its use, is the rapid diffusion and poor identification in severely anthracotic lungs (58). Furthermore, MB dyes the resected specimen which can create difficulties on histopathology assessment. IC is another dye which has been administered both percutaneously under CT-guidance and fluoroscopy but also under bronchoscopy with reported successful identification rate of 93.2% (95% CI: 90.8–95.1) (59, 60). Similarly to MB, the localization with IC is also difficult to be identified in the background of lung parenchyma with significant carbon particle deposition (55).

NIR imaging utilizes fluorescent dyes that emit in the near-infrared spectrum (700–900 nm) and offer high tissue penetration and low autofluorescence (11). Those two key features allow for sufficient contrast between the localized and non-localized area with ICG being a widely used representative of these dyes in clinical practice and medical research. ICG has increased tissue penetration at 820 nm and a greater “brightness” compared to its other NIR counterpart, MB, which emits at 700 nm and is subject to higher autofluorescence background (61). Another advantage from the use of ICG, compared to MB, is that it does not interfere with the surgical field without the use of NIR imaging system because it is otherwise non-visible to the human eye. Furthermore, ICG localized areas in patients with lung anthracosis are better visualized compared to IC or MB (55, 62). NIR imaging systems have been developed in order to detect the fluorescent dyes and aid thoracic surgeons intra-operatively in accurately detecting the localized lung nodules (63). This is perceived as a drawback for ICG because it requires specialized NIR equipment to identify the localized area. However, the availability of NIR thoracoscope cameras in thoracic departments could also be used for other parts of the procedure like the identification of intersegmental planes in anatomic sublobar lung resections (36). A second disadvantage is its poor detection of nodules that are located deep in the lung parenchyma. Even though ICG has shown better tissue penetration than the 0.5 mm of MB, its fluorescence diminshes in deep-seated nodule localization (62). Furthermore, ICG can diffuse easily in the surrounding lung parenchyma which could reduce its accuracy as a localization method. In attempt to address the two previously mentioned limitations, researchers have shown that a mixed solution of ICG with oil-based radiopaque agents, like Lipiodol can offer some benefits. That combination allows for improved localization of deep pulmonary nodules through the use of fluoroscopy and also reduces ICG diffusion (62). However, for the purposes of our study the papers that did not investigate solely the administration of ICG for localization were considered high risk of bias. This is because it was impossible to assess the true effect of ICG as a localization dye when also other techniques were in use in the same patients.

There has been a limited number of published studies that compared dye localization with other techniques. A study by Kleedehn et al. from 2016 that compared the use of hookwire with MB, showed no statistically significant difference on successful localization between the two methods (64). However, the hookwire group had more severe and frequent complications. Similarly to these findings, a more recent study by Ding et al. indicated that ICG localization also had lower complication rate compared to hookwire localization (22). Furthermore, even though the success rate was higher in the ICG group, it was also not statistically significant (22). A randomized trial from China which aims to compare ICG localization under electromagnetic navigation bronchoscopy (ENB) with percutaneous hookwire insertion could provide more evidence on the effectiveness and safety between the two techniques (NCT04182152).

To our knowledge, there are no studies available in the literature that have investigated a direct comparison between different localization dyes for lung cancer (55). For that reason, a meaningful comparison between ICG and other dyes was not possible and thus we focused our study's aim on assessing the evidence behind the effectiveness and safety amongst the different ICG localization techniques for lung cancer.

Through our systematic review, we identified three main techniques of ICG administration for lung cancer detection: percutaneous CT-guided, Intrabronchial and through peripheral Intravenous access.

CT-guided percutaneous localization of lung nodules has been widely used and is considered a well-established technique. Specifically, after using ICG as a marker, our study has shown a high reported success rate on tumor localization (96.7%) with false negative rate of merely 2.4% (26/1089). Our study also verifies the experiences from previous researchers which have shown a high incidence of complications post-localization with markers other than ICG. We found a complication rate of 15.2% (150/989) amongst patients who underwent percutaneous CT-guided localization with ICG. However, it is important to highlight that none of the cases that developed a small pneumothorax or small intra-parenchymal haematoma required any further intervention while only 11/989 patients experienced symptoms such as dyspnea, chest pain or mild hemoptysis after localization. Furthermore, the additional radiation exposure is another theoretical drawback form this procedure for which we could not collect significant evidence from our systematic review due to lack of available data.

Emerging evidence suggests that the electromagnetic navigation bronchoscopy-guided dye marking could counteract some of the problems encountered in CT-guided localization (65, 66). Our study also confirmed a high success rate of 95.4% for pre-operative localization of pulmonary nodules with ICG under bronchoscopy. We did not observe any complications related to ICG localization through this method in the literature. We assume that this can be attributed to the “single-stage” approach between localization and lung resection because the patient remains anaesthetized following localization and thus their symptoms of pain, cough or discomfort from the latter may overlap with those that the patient experiences post-operatively. Furthermore, endobronchial localization offers better access to tumors which may be located to a more central position or close to great vessels. However, effective endobronchial localization demands skillful operators who would be able to perform this procedure and train their more junior colleagues.

Finally, we reviewed the evidence behind IV administration of ICG for detection of lung malignancy. ICG is not a lung cancer specific marker but it tends to accumulate in certain tumors due to the enhanced permeability and retention (EPR) effect (67, 68). This occurs due to high permeability of the porous tumor blood vessels which results in accumulation of ICG. The retention effect is caused by the dysfunctional lymphatic system of the tumor which subsequently leads ICG to remain around the tumor's micro-environment for long enough period to make it detectable intra-operatively.

Our meta-analysis showed that IV administration of ICG has Sensitivity of 88% (95% CI: 59%–0.97%) and Specificity of 25% (95% CI: 0.04%–0.74%) in detecting lung malignancy. The wide confidence interval for Specificity reflects the small number of true negative incidences reported in the studies. Similarly, to the bronchoscopy technique, we did not find any localization-related complications among the studies that investigated IV ICG. The theoretical advantage in IV administration of ICG compared to the other two techniques is that it can identify lung nodules which may not even be detectable pre-opratively by current imaging modalities. There has been an increasing interest over the last 5 years to create NIR substances that are specific to lung cancer cells and could therefore provide more accurate tumor localization. The most widely researched are the two folate analogs EC17 and OTL38 and the oral photosensitizer 5-ALA (69).

Overall, our study shows that ICG has demonstrated high success rates as a localization dye for lung cancer. In order to accurately interpret that finding, it is important to acknowledge that some tumor-specific characteristics were incorporated in the eligibility criteria of studies that investigated different ICG localization techniques (19–48). Those frequently included the size of the nodules and their distance from the visceral pleura. In more detail, several CT-guided ICG localization studies enrolled only patients with small (<3 cm) nodules, located peripherally in the lung parenchyma at a distance of less than 3 cm from the visceral pleura (21, 23, 24, 31, 32). On the other hand, in bronchoscopy-guided ICG studies, patients were recruited when they had smaller nodules (<1 cm) on their pre-operative imaging and those were located deeper in the lung parenchyma (>1 cm from the visceral pleura) (35–38). Finally, none of the studies that investigated the IV ICG administration restricted their eligibility criteria based on tumor size or distance from the visceral pleura (19, 42–48). This discrepancy, compared to the other two localization techniques, reflects the aim of these studies, which was to identify intra-operatively even malignant nodules which were not visible on pre-operative imaging. Therefore, despite lack of general consensus, there appears to be a trend among researchers to localize lesions which are located deeper in the lung parenchyma *via* bronchoscopy whereas more superficial nodules were localized *via* CT-guidance.

The timing between administration of ICG and lung resection is essential, as the evidence suggests that it can affect the level of fluorescence in NIR imaging (62, 70). For CT- and bronchoscopy-guided ICG localization, we identified great discrepancy on time protocols between the eligible studies. Some studies reported the average time (26), others the median time (27, 31) while others only described that they proceeded with lung resection immediately after localization without providing any numerical data (24, 28, 29). The lack of homogeneity among time reporting values and the absence of analysis of the time interval between ICG localization and lung resection as a parameter that can affect successful NIR identification of ICG among the published literature does not allow for a meaningful data synthesis. For that reason, we could not accurately conclude on a specific time-interval as the ideal period before proceeding with lung resection after localization. However, it appears to be a consensus among the researchers, that lung resection is performed in less than 24 h after ICG localization. In more detail, 6/13 studies performed lung resection either immediately after CT-guided ICG localization or within 60 min (21, 22, 24, 26, 28, 29) and 5/13 within the same day (23, 25, 27, 31, 32). Similarly, from the articles that investigated bronchoscopy-guided ICG localization, in 6/12 the surgeons proceeded with lung resection immediately after localization or within 60 min (32, 33, 35, 36, 38, 40) while in 3/12 the operation was performed within 24 h (31, 32, 37). From the 8 studies that investigated the IV ICG localization, only one study administered the ICG earlier than 24 h before lung resection (43) and only one study after 24 h (47). These two studies showed the lowest successful localization estimated at 9% (43) and 73.7% respectively (47). Therefore, it appears that the consensus among most researchers (75%, 6/8) is that ICG should be administered intravenously 24 h pre-operatively (19, 42, 44, 45, 46, 48).

There are some limitations in our study's findings which derive from the design of the included studies in our review. Due to the predominantly small sample sized, single centered, retrospective, observational studies, our results should be interpreted with caution. Also, there is inevitably significant bias introduced in our results because of variations on treatment and operating protocols among the different units. Given that there are no available data in the literature from meaningful comparison between different ICG localization techniques or different dyes, we were not able to perform any analysis on this topic. Furthermore, is important to mention that the absence of allergic reactions from our findings could have been influenced by the exclusion of patients with iodine allergies from the included studies.

Nevertheless, our study offers an updated and thorough review on how effective is ICG in detecting pulmonary malignancies. Our findings will complement further research projects that will advance evidence-based medicine in thoracic surgery. Large prospective multicenter studies are required in order to safely validate our results. Presumably, a 2-arm, multicentre, randomised, surgeon-blinded, parallel design clinical trial that will compare CT- with bronchoscopy-guided ICG localization of lung nodules will provide meaningful data. The two groups should be stratified according to nodule size and their distance from the visceral pleura which could allow for further sensitivity analysis of their effect on successful ICG localization.

The design of standardized localization protocols will be a key component in future research projects in thoracic surgery.

## Data Availability

The original contributions presented in the study are included in the article/Supplementary Material, further inquiries can be directed to the corresponding author/s.
