# Supplementary figures and images for: How effective is indocyanine green (ICG) in localization of malignant pulmonary nodules? A systematic review and meta-analysis
Source: Front Surg. 2022 Jul 25;9:967897. doi: 10.3389/fsurg.2022.967897 (PMC9357917; doi:10.3389/fsurg.2022.967897)

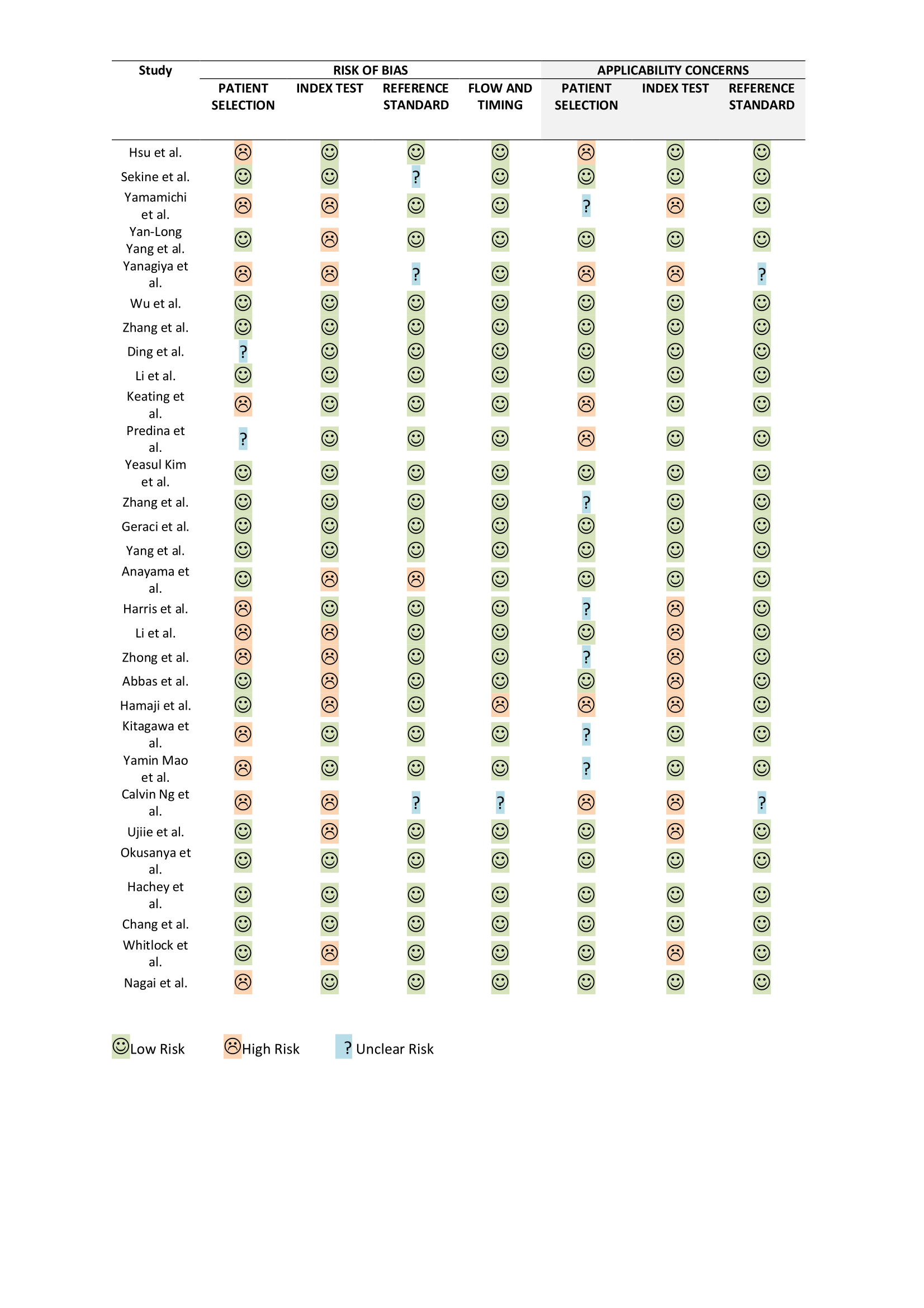

Supplement: Supplementary file 1 [file Image_1_v1.tiff]
